# Blockade of Vascular Endothelial Growth Factor Receptor 1 Prevents Inflammation and Vascular Leakage in Diabetic Retinopathy

**DOI:** 10.1155/2015/605946

**Published:** 2015-03-03

**Authors:** Jianbo He, Hong Wang, Ying Liu, Wen Li, Dorothy Kim, Hu Huang

**Affiliations:** ^1^Guangxi Tumor Hospital & Institute, Nanning, Guangxi 530021, China; ^2^Department of Ophthalmology, the Provincial Hospital Affiliated to Shandong University, Jinan, Shandong 250021, China; ^3^Changsha Aier Eye Hospital, Changsha, Hunan 410015, China; ^4^Aier School of Ophthalmology, Central South University, Changsha, Hunan 410015, China; ^5^Wilmer Eye Institute, Johns Hopkins University School of Medicine, M017 Robert H. and Clarice Smith Building, 400 N Broadway, Baltimore, MD 21287, USA

## Abstract

Diabetic retinopathy (DR) is a leading cause of blindness in working age adults. The objective of this study is to investigate the effects of vascular endothelial growth factor receptor 1 (VEGFR1) blockade on the complications of DR. Experimental models of diabetes were induced with streptozotocin (STZ) treatment or Insulin2 gene mutation (Akita) in mice. Protein expression and localization were examined by western blots (WB) and immunofluorescence (IF). mRNA expression was quantified by PCR array and real-time PCR. The activity of VEGFR1 signaling was blocked by a neutralizing antibody called MF1. Vascular leakage was evaluated by measuring the leakage of [^3^H]-mannitol tracer into the retina and the IF staining of albumin. VEGFR1 blockade significantly inhibited diabetes-related vascular leakage, leukocytes-endothelial cell (EC) adhesion (or retinal leukostasis), expression of intercellular adhesion molecule- (ICAM-) 1 protein, abnormal localization and degeneration of the tight junction protein zonula occludens- (ZO-) 1, and the cell adhesion protein vascular endothelial (VE) cadherin. In addition, VEGFR1 blockade interfered with the gene expression of 10 new cytokines and chemokines: cxcl10, il10, ccl8, il1f6, cxcl15, ccl4, il13, ccl6, casp1, and ccr5. These results suggest that VEGFR1 mediates complications of DR and targeting this signaling pathway represents a potential therapeutic strategy for the prevention and treatment of DR.

## 1. Introduction

Diabetes mellitus (DM) is a widespread disorder with a prevalence of about 285 million in 2010 and predicated increase to 439 million by 2030 [1]. DR is one of the most common complications of DM and affects about 93 million people worldwide [2]. Clinically, DR is divided into two forms: nonproliferative DR (NPDR) and proliferative DR (PDR). Diabetic macular edema (DME) and retinal neovascularization are the two main causes of visual impairment and blindness in patients with DR [3]. Its pathological features include increased vascular permeability or breakdown of BRB, neovascularization (NV), capillary nonperfusion, endothelial cell damage, and apoptotic cell death of retinal neurons, endothelial cells, and pericytes. The early events, such as endothelial cell-leukocyte adhesion (or retinal leukostasis) and oxidative stress, contribute to these clinical and pathological characteristics in DR.

VEGFR1 has been reported to play various roles in the vascular development, angiogenesis, cell survival, and inflammation. First of all, as a VEGF-A trap or sink, VEGFR1 (mainly soluble VEGFR1 or FLT1), has been characterized as a negative regulator in both embryonic and postnatal vascular development [4, 5]. Secondly, VEGFR1 has been shown to be a positive mediator of pathological angiogenesis in the experimental models of some primary tumors and wet age-related macular degeneration (AMD) [6]. Thirdly, VEGFR1 has been reported to promote cell survival under some stress conditions. For instance, in the oxygen-induced retinopathy (OIR) model, VEGFR1 activation by placental growth factor (PlGF) could prevent vessel obliteration or degeneration during the hyperoxia phase, thereby preventing the subsequent vessel proliferation during the hypoxia phase [7]. In addition, VEGFR1 signaling plays a role in regulating the chemotaxis of inflammatory cells [8–10].

The functions of VEGFR1 vary depending on the pathophysiological microenvironment, the type of ligand that binds (PlGF, VEGF-A, or VEGF-B), and the formation of VEGFR1-VEGFR2 heterodimers. Whether the VEGFR1 plays a role in the pathogenesis of DR remains unknown. In the present study, we address this question by blocking the VEGFR1 activity with an antibody called MF1. This VEGFR1-specific antibody has been previously reported by us and other investigators [8, 10, 11]. We found that VEGFR1 blockade prevented vascular leakage and retinal leukostasis, degeneration, and disorganization of the tight junction protein zonula occludens- (ZO-) 1 and the adhesion molecule vascular endothelial (VE) cadherin in DR.

## 2. Methods

### 2.1. Mouse Models of Diabetes

All animals were used in accordance with the approved protocols by the Institutional Animal Care and Use Committee of Johns Hopkins University School of Medicine and the guidelines of the ARVO Statement for the Use of Animals in Ophthalmic and Vision Research. Two mouse models of diabetes were used: one was streptozotocin- (STZ-) induced method and the other was an Akita diabetic mouse, both of which were described by our previous paper [12].

### 2.2. Administration of Anti-VEGFR1 Antibody

The monoclonal antibody MF1 was used to block VEGFR1 activity. 50 mg antibody per 1 kg mouse body mass was intraperitoneally (IP) injected three times per week as we performed previously [6]; rat IgG was used as the treatment control. This dose was used because it showed high efficacy in inhibiting pathological angiogenesis and infiltration of inflammatory cells in the mouse models of laser-induced CNV and oxygen-induced retinopathy (OIR) [6, 11]. In the present study, the preventative approach was implemented: the treatments started shortly after the onset of hyperglycemia and long before the occurrence of diabetic complications.

### 2.3. Western Blots (WB) and Quantification Analysis

Retinas were homogenized in an ice-cold lysis buffer [150 mM NaCl, 20 mM Tris (pH 7.4), 2 mM ethylenediaminetetraacetic acid (EDTA), 1% Triton X-100, and complete mini EDTA-free protease inhibitor] by sonication for 3–5 seconds, were incubated for 30 minutes, and were centrifuged at 14,000 g at 4°C for 10 minutes. Supernatant was collected and protein concentrations were measured by the bicinchoninic acid (BCA) method. Twenty-to-thirty micrograms of protein were electrophoresed on 4%–15% gradient SDS PAGE gels and then transferred to nitrocellulose membranes. After blocking with 5% bovine serum albumin (BSA) or 5% nonfat milk for 1 hr, protein blots were incubated with primary antibodies in the cold room overnight, followed by horseradish peroxidase- (HRP-) conjugated secondary antibodies (1 : 2,000–5,000) and developed with chemiluminescence reagents (Pierce Technology Co., Holmdel, NJ). The optical density (OD) of each protein band was determined with Image J software according to the user's instructions. The OD of VEGFR1 protein was normalized by dividing with that of *β*-actin of the same protein sample. The normalized OD ratio of the diabetic sample to the nondiabetic sample was designated as fold changes.

### 2.4. Immunofluorescence (IF) Staining and Quantification

10 *μ*m cryosections were blocked/permeabilized with PBS buffer containing 0.25% Triton X-100 and 10% goat serum for one hour and incubated at 37°C for two hours or overnight at 4°C with primary antibodies: rat anti-VEGFR1 (ImClone, Somerville, NJ), mouse anti-ICAM-1 (DSHB, Iowa city, IA), rabbit anti-albumin (Nordic, Capistrano Beach, CA), rat anti-CD31 (Novus Biologics, Littleton, CO), mouse anti-VE cadherin (DSHB), and rat anti-ZO-1 (DSHB). After a rinse was repeated 3 times with PBS buffer, specimens were incubated with appropriate secondary antibodies at 1000 dilution. For double-labeling IF staining of albumin and CD31/PECAM1, the cocktail of two primary antibodies from distinct species was applied, and the appropriate secondary antibodies conjugated with Alex fluo 594 or 488 were used to visualize the staining with fluorescence microscopy. DAPI was used for counter-staining. In the case of mouse primary antibodies, the anti-mouse secondary antibody was preincubated with 0.03 mg/ml normal mouse IgG (Invitrogen, Cat no: 10400C) to prevent its association with the endogenous mouse IgG, thus minimizing background staining.

For the quantitative comparison of IF images, the specimens and images were prepared to eliminate the errors caused by variations with particular care, as described previously [13]. Briefly, the eye samples of control and experimental groups (*n* = 4 each group) were cryopreserved in the same module with the superior quarter up. The cryosections including optical nerves were collected for immunostaining. The procedures were therefore parallel with the treatment and control groups. A Zeiss Axioplan2 fluorescent microscopy was used to acquire IF Images with Axion 4 software. The IF-stained specimens were used for quantification within 1 week. The fluorescence intensity and area of IF images were quantified by Image J software. The IF-positive areas were first identified as a region of interest (ROI) by running Image > Adjust > Threshold. The mean intensity and area of ROI were then determined by running Analyze/Analyze particles. The results were averaged from the 4 cryosections and then expressed as mean ± SD per section (10 *µ*m).

### 2.5. PCR Array and Real-Time Quantitative (Q) PCR

Total RNA was prepared from the retinas of 5~6-month-old Akita diabetic male mice (with 4~5 months' diabetic duration) and the nondiabetic littermate male mice by using Trizol agents based on the manufacturer's manual. cDNA was synthesized with SuperScript III First-Strand Synthesis System (Invitrogen). The mouse inflammatory cytokine PCR array (Cat no: 330231 PAMM-011A, SABiosciences), containing 84 important cytokine, chemokine, or receptor genes, was used to screen the novel genes whose expression was affected by diabetes. Real-time QPCR was used to quantify the gene expression alterations of cytokine and chemokine due to VEGFR1 blockade. Primers were designed as follows: the mRNA nucleotide sequences were obtained from the Unigene database by searching their gene symbols. The achieved sequences were aligned with the mouse genome by using an online Blat program to identify the locations of exons. The primers were designed with the primer3 software. To circumvent the contaminations from genomic DNA with intron sequences, the forward and reverse primers were located in the two or more distinct exons (see Supplementary Table 1 for sequence information available online at http://dx.doi.org/10.1155/2015/605946). Quantification of real-time PCR was performed as we previously described [6].

### 2.6. The Quantitative BRB Assay

The quantitative BRB assay was performed according to a previously described technique [12] with some modifications. Mice were sedated as above and given an IP injection of 1 *μ*Ci/gram body weight of [^3^H]-mannitol. One hour after injection, the mice were sedated and retinas from the experimental and control eyes were rapidly removed. The posterior portion of the globe was firmly grasped with forceps and a razor blade was used to cut across the cornea and extrude the lens, vitreous, and retina. Retinas were dissected free from the lens, vitreous, and any RPE that was extruded and were placed within preweighed scintillation vials within 30 seconds of sacrifice. The thoracic cavity was opened and the left superior lobe of the lung was removed, blotted free of excess blood, and placed in another preweighed scintillation vial. A left dorsal incision was made and the retroperitoneal space was entered without entering the peritoneal cavity. The renal vessels were clamped with forceps and the left kidney was removed, cleaned of fat, blotted, and placed into a preweighed scintillation vial. Superficial liquid was allowed to evaporate over 20 min from the open vials. The vials containing the tissue were weighed and tissue weights were calculated and recorded. 1 ml of NCSII solubilizing solution was added to each vial and the vials were incubated overnight in a 50°C water bath. Solubilized tissue was brought to room temperature (RT) and decolorized with 20% benzoyl peroxide in toluene in a 50°C water bath. The vials were brought to RT and 5 ml of Cytoscint ES and 30 *μ*l of glacial acetic acid were added. The vials were stored for several hours in darkness at 4°C to eliminate chemiluminescence. Radioactivity was counted with a LS 6500 Liquid Scintillation Counter (Beckman, Brea, CA). The CPM/mg tissue was measured for the lung, kidney, and experimental and control retinas. Retina/lung and retina/kidney ratios were calculated and compared.

### 2.7. Retinal Leukostasis

Mice were first anesthetized with excess carbon dioxide, the descending aorta was clamped, and the right atrium was cut. The mice were perfused with 5 ml PBS to remove erythrocytes and nonadherent leukocytes, followed by perfusion of fluorescein-conjugated Con-A to label the adherent leukocytes. Another PBS perfusion was used to flush out unbound fluorescein. Retinal flat mounts were prepared to assess leukostasis. The eyes were harvested and fixed for more than 1 hour with phosphate-buffered formalin. The cornea and lens were removed and, under a stereomicroscope (Stemi 2000C; Carl Zeiss Meditec, Inc., Thornwood, NY), the entire retina was carefully dissected from the eye cup and rapidly cut from the edge to the equator in all four quadrants and was flat-mounted with the photoreceptors facing upward. Leukocytes adherent to the vessel walls were labeled with fluorescein, and leukocytes within the vessels of each retina were counted under an epifluorescence microscope (Axio-pan2; Carl Zeiss Meditec, Inc.) by an investigator masked to the nature of the specimen. The counting began at the optic disc. The vessel at the 12-o'clock position was first examined from the optical disk to the edge of vasculature, and the focus was adjusted as necessary to include all the arteries, veins, and capillaries in the field. This process was repeated in a clockwise direction for each vessel radiating from the optic disc, so the total number of adherent leukocytes in all of the vessels of the retina was counted.

### 2.8. Statistical Analysis

The Mann-Whitney test was used for the statistical analyses of comparisons between groups: diabetic retina versus nondiabetic retina and antibody treatment versus rat IgG treatment. *P* < 0.05 was designated as being statistically significant.

## 3. Results

### 3.1. Increased Expression of VEGFR1 Protein in Diabetic Mouse Retina

**Figure 1:**
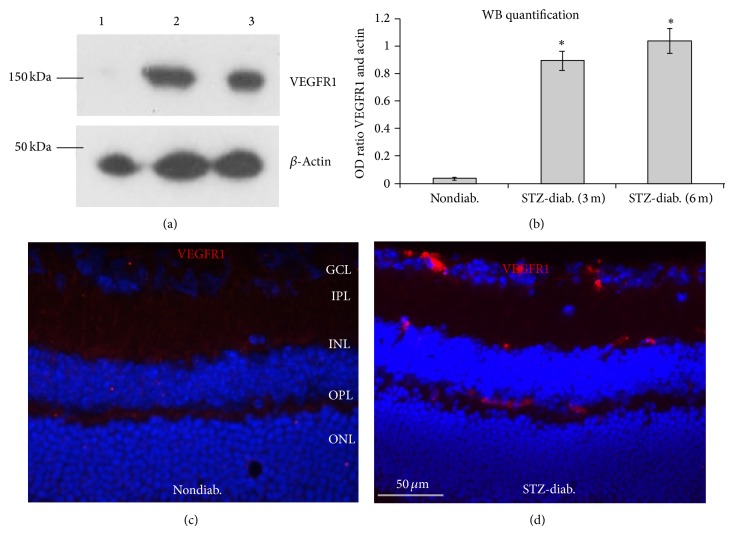
Increased expression of VEGFR1 protein in the retina of STZ-induced mice. The STZ-induced diabetic mice with 3 or 6 months of diabetes duration were used for this analysis. (a) Western blots (WB) results. *β*-Actin was used for loading control. (b) The WB quantification results (*n* = 4). ^*^ *P* < 0.05 versus nondiabetic controls. ((c) and (d)) IF results showed that VEGFR1 protein localized in the blood vessels of the diabetic mouse retina (d) but was not detected in the nondiabetic control mouse retina (c). GCL: ganglion cell layer; IPL: inner plexiform layer; INL: inner nuclear layer; OPL: outer plexiform layer; ONL: outer plexiform layer. Scale bar: 50 *μ*m.

Increased expression of VEGFR1 has been previously shown in the retinal vasculatures of diabetic rat and human [14–16], but whether its expression is also increased in diabetic mice remains unknown. We therefore examined VEGFR1 protein expression in diabetic mouse retina. The WB results showed that VEGFR1 expression was increased in STZ-induced diabetic mice (3-month diabetes duration) compared with nondiabetic controls; and IF staining further showed its vascular localization (Figure 1). In Akita diabetic mice (both 6- and 8-month diabetes duration), a robust and specific protein band of approximately 180 KDa was detected by anti-VEGFR1 antibody, but no or weak protein band was detected in nondiabetic mice (Supplementary Figures 1(a) and 1(b)). Similarly, IF staining showed that there was no or barely detectable immunoreactivity for VEGFR1 in nondiabetic mice (Supplementary Figure 1(c)); whereas IF intensity was evidently elevated in diabetic mice with an apparent vascular localization (Supplementary Figure 1(d)). Quantification showed that VEGFR1 protein expression was significantly upregulated by diabetes (Supplementary Figures 1(e) and 1(f)).

### 3.2. VEGFR1 Blockade Inhibits Retinal Leukostasis in DR

**Figure 2:**
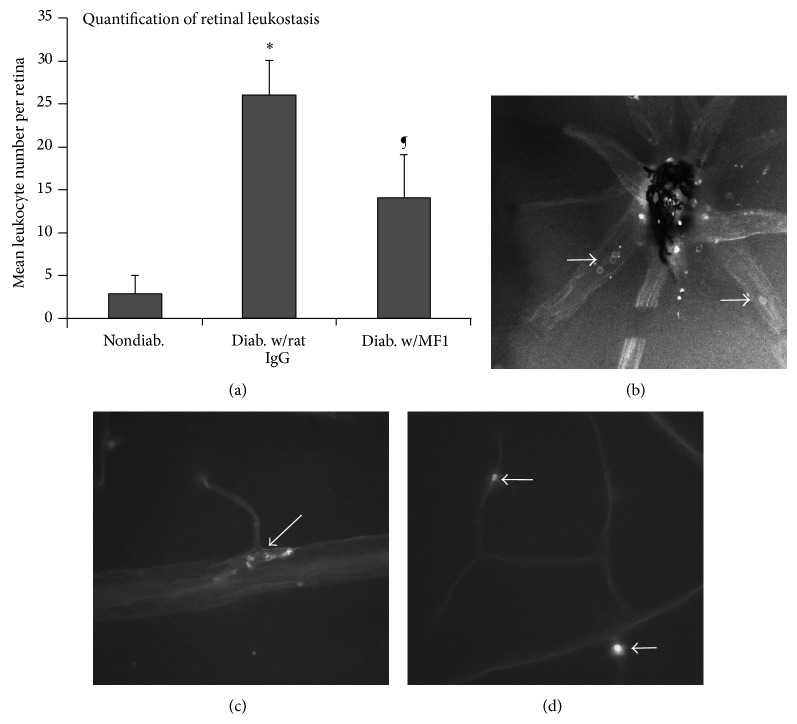
Inhibition of diabetes-caused retinal leukostasis by VEGFR1 blockade. The STZ-induced diabetic mice with two-week diabetes duration were used for this analysis. (a) The quantification showed that the number of leukocytes adhering to the retinal vasculature was significantly increased in the retinas of the diabetic mice compared with the nondiabetic mice but decreased due to VEGFR1 blockade. The total time of MF1 injection was 6 (3x/week). The results were expressed as the mean number ± SD (*n* = 5). ^*^ *P* < 0.05 versus nondiabetic mice. ¶ versus diabetic mice treated with rat IgG. ((b)–(d)) Fluorescein-labeled leukocytes in the retinal vasculature were photographed and converted to black-and-white images, which showed the localization of leukocytes in larger arteries and veins ((b) and (c)) and smaller capillaries (d). Arrows indicated the leukocytes.

Six-week-old STZ-induced diabetic mice (about 2-week diabetes duration) and nondiabetic mice of the same age were used for this analysis. Similar to our previous results [12], 2-week diabetes duration caused a significant increase of leukocytes in comparison with the nondiabetic mice (Figure 2(a)). We observed that the leukocytes presented in capillary as well as arteries and veins in the diabetic retina (Figures 2(b)–2(d)). It was obvious that the cells in the capillary vessels transverse the whole lumen space, thus becoming an obstacle for blood flow. On some occasions, the cells aggregated at the root of branches from the larger vessels to the smaller capillaries (Figure 2(c)), preventing the blood from circulating into the capillaries.

### 3.3. VEGFR1 Blockade Inhibits ICAM-1 Upregulation in DR

**Figure 3:**
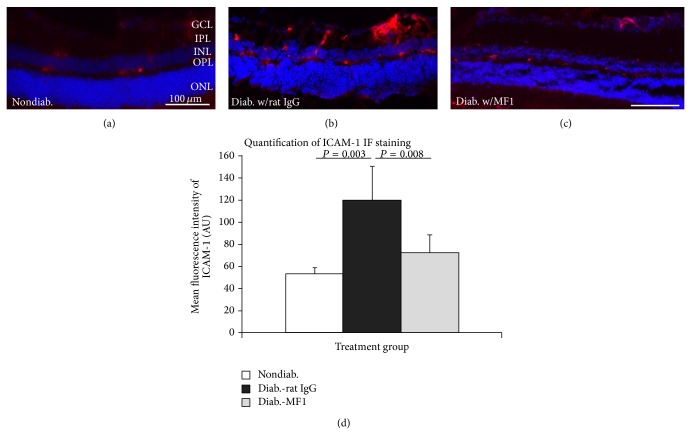
Inhibition of diabetes-caused upregulation of ICAM-1 by VEGFR1 blockade. ((a)–(c)) Immunofluorescence (IF) results from the nondiabetic mice (a) and the STZ-induced diabetic mice that were treated with rat IgG (b) and MF1 (c). (d) Quantification of IF images showed the statistically significant difference. The results were averaged from 4 mice (*n* = 4).

ICAM-1 plays an important role in retinal leukostasis. We examined the effects of VEGFR1 blockade on ICMA-1 expression. In nondiabetic mice, ICAM-1 immunoreactivity was generally weak in the majority of blood vessels; very few blood vessels showed strong immunoreactivity (Figure 3(a)). ICAM-1 immunoreactivity was strong in all ICAM-1(+) blood vessels in diabetic mice (Figure 3(b)). ICAM-1 Immunoreactivity was weaker in the MF1-treated mice than in rat IgG-treated ones. The signal intensity of ICAM-1 immunoreactivity was faint in the majority of blood vessels but strong only in very few blood vessels (Figure 3(c)). MF1 treatment apparently resulted in a reduction of ICAM-1 immunostaining intensity compared with the mice treated with rat IgG in the vasculature. Quantification further showed the significant differences among the three treatment groups (Figure 3(d)).

### 3.4. VEGFR1 Blockade Inhibits Vascular Leakage in DR

**Figure 4:**
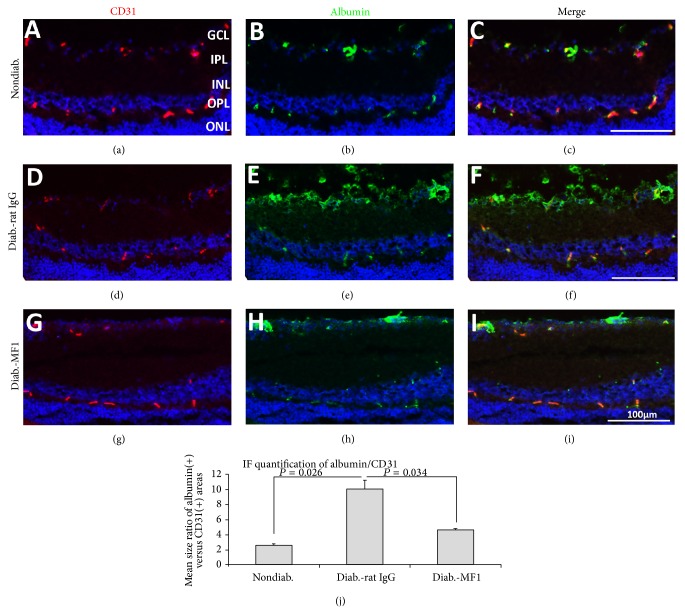
Inhibition of diabetes-caused albumin leakage by VEGFR1 blockade. ((a)–(i)) The representative immunofluorescence (IF) images were taken from the nondiabetic control mice ((a)–(c)) and the STZ-induced diabetic mice that were treated with rat IgG ((d)–(f)) or MF1 ((g)–(i)). The diabetic duration was 3 months (or 13 weeks) and the total time of rat IgG or MF1 treatment was 39 (3 times/week). Scale bar: 100 *μ*m. (j) Quantification showed the size ratio of albumin (+) areas versus CD31 (+) blood vessel areas in the three groups. The results were averaged from 4 mice (*n* = 4).

**Figure 5:**
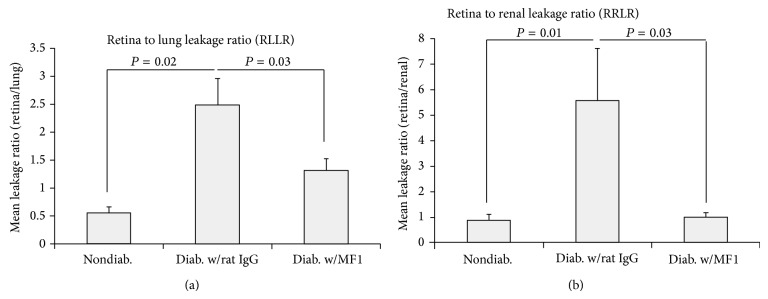
Inhibition of diabetes-caused BRB breakdown by VEGFR1 blockade. Nondiabetic mice and STZ-induced diabetic mice that were treated with rat IgG or MF1 were used for quantitative BRB assay. The diabetic duration was 3 months (or 13 weeks) and the total time of rat IgG or MF1 treatment was 39 (3 times/week). The CPM reading of retina, lung, or kidney was normalized by its own tissue mass (CPM/mg). RLLR (retina to lung leakage ratio) and RRLR (retina to renal leakage ratio). The results were averaged from 5 mice (*n* = 5). ^*^ *P* < 0.05.

Albumin staining was used to examine the sites of vascular leakage and the effects of VEGFR1 blockade. Double-labeling immunofluorescence staining showed that the albumin protein colocalized well with the endothelial cell marker CD31/PECAM-1 in nondiabetic mice, suggesting its localization in the blood vessels without leakage (Figures 4(a)–4(c)). By contrast, albumin immunoreactivity was not restrained to the CD31 (+) vessel areas in the diabetic mice treated with Rat IgG (Figures 4(d)–4(f)). In the diabetic mice treated with MF1, the staining results showed that albumin protein colocalized largely with CD31 (Figures 4(g)–4(i)), which was very similar to those from the nondiabetic mice. Quantification showed the intensity ratio of albumin and CD31 immunoreactivity was significantly different among the three groups (Figure 4(j)). In addition, the degree of vascular leakage was further measured by a quantitative BRB assay. The results showed that retina to lung leakage ratio (RLLR) and retina to renal leakage ratio (RRLR) were significantly reduced by VEGFR1 blockade (Figure 5).

### 3.5. VEGFR1 Blockade Preserves BRB Integrity in Diabetic Retina

**Figure 6:**
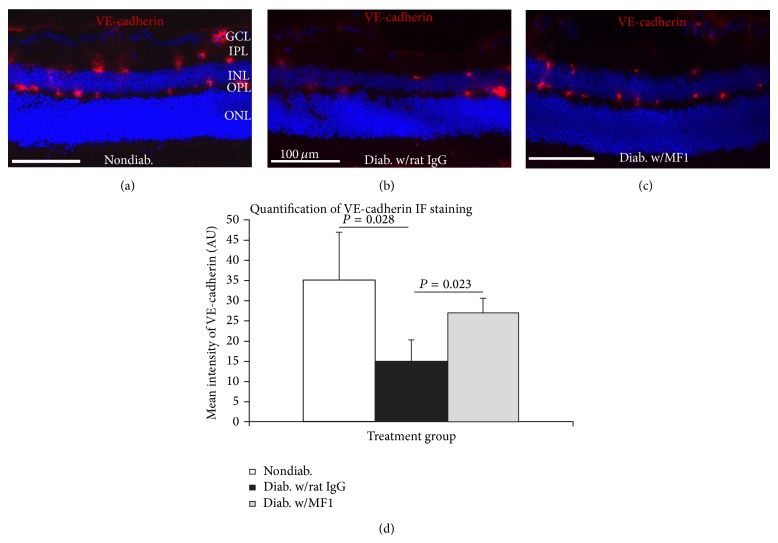
Prevention of diabetes-caused abnormal localization and degeneration of VE cadherin by VEGFR1 blockade. ((a)–(c)) The representative immunofluorescence (IF) images were taken from the nondiabetic C57BL6 mice (a) and the STZ-induced diabetic mice that were treated with rat IgG (b) or MF1 (c). The diabetic duration was 3 months (or 13 weeks) and the total time of rat IgG or MF1 treatment was 39. Note the pattern difference: the “foggy and cloudy” pattern for rat IgG treatment group and the “clear and vessel-like” pattern for the nondiabetic and MF1 treatment groups. GCL: ganglion cell layer; IPL: inner plexiform layer; INL: inner nuclear layer; OPL: outer plexiform layer; ONL: outer plexiform layer. Scale bar: 100 *μ*m. (d) Quantification showed the intensity difference in the three groups. The results were expressed as the mean ± SD (*n* = 4).

We investigated the effects of VEGFR1 blockade on the expression and localization of zonula occludens- (ZO-) 1 and vascular endothelial (VE) cadherin, which are the tight junction and adhesion proteins of BRB. VE cadherin immunoreactivity was very strong and showed a very sharp and blood vessel-like staining pattern in nondiabetic mice, whereas the staining looked foggy and cloudy in diabetic mice. In MF1, the staining showed “clear and vessel-like” patterns similar to those observed in the nondiabetic retinas (Figures 6(a)–6(c)). The quantification showed that the fluorescence intensity of VE cadherin was significantly lower in diabetic mice than nondiabetic mice and MF1 treatment significantly increased its expression compared with the rat IgG control treatment.

**Figure 7:**
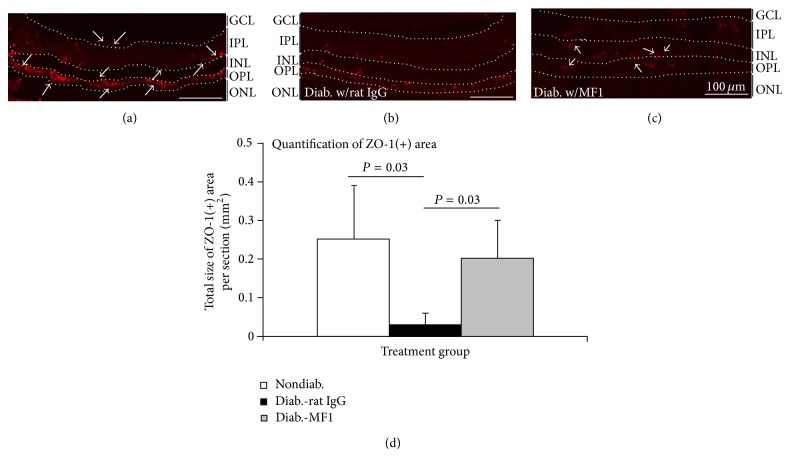
Prevention of diabetes-caused abnormal localization and degeneration of ZO-1 by VEGFR1 blockade. ((a)–(c)) The representative immunofluorescence (IF) images were taken from the nondiabetic mice (a) and the STZ-induced diabetic mice that were treated with rat IgG (b) or MF1 (c). The diabetic duration was 3 months (or 13 weeks) and the total time of rat IgG or MF1 treatment was 39 (3 times/week). The dash lines separate the retinal layers. GCL: ganglion cell layer; IPL: inner plexiform layer; INL: inner nuclear layer; OPL: outer plexiform layer; ONL: outer plexiform layer. Scale bar: 100 *μ*m. (d) Quantification showed the difference of mean size of ZO-1(+) areas in the three groups. The results were expressed as mean ± SD (*n* = 4).

In nondiabetic mice, IF staining of ZO-1 showed a “ring and string” pattern, which may reflect that ZO-1 proteins are well localized in the walls of both large retina arteries and veins as well as in the small capillaries (Figure 7(a)); in diabetic mice, the staining looked like short rod and stick, indicating that ZO-1 proteins were displaced in the large vessels and were diminished in the capillaries (Figure 7(b)). In the antibody treated mice, the lumen structures were hardly detected. However, the capillary structures were visible in some ZO-1(+) vessels. Quantification showed that the mean size of the ZO-1(+) vessels was significantly different (Figure 7(d)).

### 3.6. VEGFR1 Blockade Influences Gene Expression of Cytokine/Chemokine

**Figure 8:**
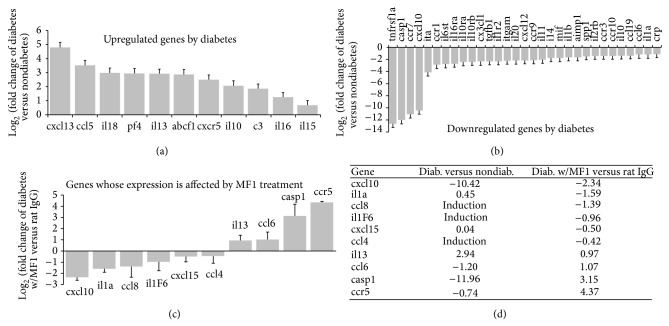
Influence of diabetes and VEGFR1 blockade on gene expression of cytokines and chemokines. The differentially expressed genes between diabetic and nondiabetic retina were identified by cytokine and chemokine PCR arrays *n* = 3 each group. The STZ-induced diabetic mice (3 months diabetes duration) and nondiabetic mice of the same age were used for this experiment. (a) Upregulated genes by diabetes. (b) Downregulated genes by diabetes. (c) Genes whose expression was affected by MF1 treatment were quantified by real-time PCR (*n* = 4 each group). (d) The table showed the fold changes of gene expression between diabetic and nondiabetic mice and between MF1 treatment and rat IgG treatment. The results were the mean value of log_2_ (fold changes) ± SD (*n* = 4). Induction means that the gene was detected only in the diabetic mouse retina but not in the nondiabetic mouse retina.

We investigated the influences of VEGFR1 blockade on the expression of 84 important cytokine and chemokine genes that were contained in an array plate. In total, the expression of 67 genes was found in the retina. The expression of 9 genes was detected only in the diabetic retinas but not in the nondiabetic retinas: cxcr2, cxcl9, itb, ccl8, il1f6, ccl4, cxcl10, cxcl11, and ccl20. Expressions of 11 genes were upregulated by diabetes: cxcl13, ccl5, il18, pf4, il13, abcf1, cxcr5, il10, c3, il16, and il15 (Figure 8(a)), and expressions of 31 genes were downregulated by diabetes: such as tnfrsf1a, casp1, ccr7, and cxcl10 (Figure 8(b)). Furthermore, the genes whose expressions were altered by diabetes were selected to investigate the effects by VEGFR1 blockade. Expressions of 10 genes were found to be altered by VEGFR1 blockade compared with rat IgG control: cxcl10, il10, ccl8, il1f6, cxcl15, ccl4, il13, ccl6, casp1, and ccr5 (Figures 8(c) and 8(d)).

## 4. Discussion

In this study, we first found that VEGFR1 protein expression was increased more than 10-fold in the retinas of diabetic mice compared with nondiabetic mice. This result is consistent with previous reports that showed upregulation of VEGFR1 expression in several other species, such as rat and human [14–16]. We then found that VEGFR1 blockade prevented vascular leakage and retinal leukostasis, degeneration, and disorganization of the tight junction protein ZO-1 and the adhesion molecule VE cadherin in DR. We started treating the mice shortly after the onset of hyperglycemia and long before the occurrence of any complications of DR. Therefore, the results of this study display the preventative effects of VEGFR1 blockade on vascular complications DR. In order to know whether VEGFR1 blockade can also be therapeutically effective or reverse the disease progression, it would be necessary to treat mice at a later stage when complications of diabetes have already taken place because DR is a chronic ischemic and inflammatory disorder and many diabetes-associated complications start appearing at relatively later stages. Finally, we found that VEGFR1 blockade inhibited gene expression of inflammatory marker ICAM-1 and other cytokine and chemokine genes, providing further evidence that VEGFR1 signaling is involved in the regulation of inflammation in DR.

Our findings suggest that targeting VEGFR1 may be a complementary strategy to anti-VEGF therapy, which is widely used to treat vascular disorders, including, age-related macular degeneration (AMD), DR, and DME. This likelihood is also supported from the outcomes from other studies. For instance, VEGFR1 signaling has been suggested to be associated with the diseased cells and tissues more than the normal ones, so inhibiting its activity likely results in less side effects than anti-VEGF therapy [17]. The potency of blocking VEGFR1 appears comparable to that of anti-VEGF antagonists in the experimental models of cancer and ocular angiogenesis [6, 18]. VEGFR1 has been shown to inhibit infiltration of inflammatory cells or orchestrate interplay of VEGF-A, PlGF, and VEGFR2 [11, 19]. In addition, another possible alternative strategy could target the upstream regulators of VEGF, such as prolyl hydroxylase (PHD) and hypoxia inducible factor (HIF) [20]. Because they regulate a broad spectrum of gene expressions, inhibition of their activities may lead to a normalization of vasculature instead of impairment and degeneration.

DR is a complex disease involving a multiple of biochemical and molecular changes. In addition to VEGF and its receptor signaling, a number of other molecules and signaling pathways are implicated in the pathogenesis of DR, such as protein kinase C- (PKC-) *β*/-*δ* [21, 22], transforming growth factor- (TGF-) *β* [23], tumor necrosis factor- (TNF-) *α* [12], *β*-catenin (a key regulator of Wnt pathway) [24], cyclooxygenase- (COX-) 2 [25], NADPH oxidase (NOX)2 [26], arginase-1/-2 [27], aldose/aldehyde reductase (AR) [28], nuclear factor kappa-light-chain-enhancer of activated B (NF-*κ*B) cells [29], forkhead box protein O1 (FOXO1) [30], inducible nitric oxide synthase (iNOS) [31], and altered* O*-GlcNAc signaling [32]. Understanding these underlying molecular cascades helps develop therapeutic strategies and ushers in clinical trials or applications, especially targeted therapy. The most successful example is VEGF, which was originally discovered as a vascular permeability and mitogen factor [33, 34]. The two VEGF antagonists Lucentis (ranibizumab) and Eylea (aflibercept) have been approved by US Food and Drug Administration to treat the patients with DME. Despite the success, special attention must be paid to the safety and efficacy of treatments in patients, because they do not provide a “cure” and not all patients respond; there are potential side effects since VEGF is a survival factor for choriocapillary, retinal neuron, and RPE [35–37]. Therefore, Search for more optimal therapeutic strategies is necessary for the improvement of anti-VEGF therapy.

In summary, the results from this study suggest that VEGFR1 is involved in the pathogenesis of DR and a potential target for this disease. The development of VEGFR1 inhibitors is clinically relevant because they could be potentially used for alternative and/or combinatory treatments to anti-VEGF therapy.

## Supplementary Material

Supplementary Table 1: The primer information for real-time PCR. The gene sequence was achieved from Genbank through the accession number. The oligonucleotide sequences were selected by primer3 software. Each primer pair amplified the unique PCR product as indicated. Supplementary Figure 1: The description was provided in the manuscript already.
