# Supplementary material for: Blockade of Vascular Endothelial Growth Factor Receptor 1 Prevents Inflammation and Vascular Leakage in Diabetic Retinopathy
Source: J Ophthalmol. 2015 Mar 3;2015:605946. doi: 10.1155/2015/605946 (PMC4363713; doi:10.1155/2015/605946)

Supplementary Table 1. Primer sequence of cytokine genes.

Gene	Access No.	Forward	Reverse	Length
ltb	NM_008518.2	tatcactgtcctggctgtgc	tcctggaagcattggatctc	315
casp1	BC008152.1	aaagaaacatgcgcacacag	cctctttgccctcaggatct	266
cxcl9	BC003343.1	tccttttgggcatcatcttc	agtccggatctaggcaggtt	126
cxcr2	NM_009909.3	gctgcctcactttcttccag	agggcatgccagagctataa	2919
ccl8	NM_021443.3	acgctagccttcactccaaa	tctggaaaaccacagcttcc	111
cxcl11	NM_019494.1	gctgctgagatgaacaggaa	actttgtcgcagccgttact	126
il1f6	NM_019450.3	tggcagctcagaaacaacat	ggcatgggagcaaggtaata	139
ccl4	NM_013652.2	gccctctctctcctcttgct	gtctgcctcttttggtcagg	114
cxcl10	NM_021274.1	aagtgctgccgtcattttct	gtggcaatgatctcaacacg	126
ccl20	NM_016960.2	cttgctttggcatgggtact	aggaggttcacagccctttt	114
il1f8	AY071842.1	ctttggaccaaggcaatgat	ttccagtcaggacccatacc	107
cxcr3	NM_009910.2	gcaagttcccaaccacaagt	tctcgttttccccataatcg	1506
cxcl15	NM_011339.2	tcctgctggctgtccttaac	taattgggccaacagtagcc	129
il1b	NM_008361.3	tgtgaaatgccaccttttga	tgtcctcatcctggaaggtc	201
il13	NM_008355.3	cagctccctggttctctcac	ggaatccagggctacacaga	104
ccl6	M58004.1	aggctggcctcatacaagaa	tcccctcctgctgataaaga	111
ccr5	D83648.1	tcctagccagaggaggtgag	agccgcaatttgtttcacat	2823
il1a	NM_010554.4	gcaacgggaagattctgaag	tgacaaacttctgcctgacg	173
il10	M37897.1	tgctatgctgcctgctctta	tcatttccgataaggcttgg	152
cxcr5	NM_007551.2	catgggctccatcacataca	gtgcctctccaggattacca	2514

Supplementary Figure 1. Increased expression of VEGFR1 protein in Akita diabetic mouse retina. (A & B) Western blots (WB) results. The anti-VEGFR1 antibody MF1 detected a protein band of approximately 180KDa in the retinas of 8~9-month-old (lane 2 in A & left lane 2 in B) and 5~6-month-old (left Lane 3 in B) Akita diabetic mice but did not (lane 1 in A) or weakly (left lane 1 in B) in the retinas of the non-diabetic control mice. For protein loading controls, the anti-β-actin antibody was mixed with MF1 and probed the protein blots simultaneously (A) or it separately probed the striped protein blots (B). (C & D) IF results showed VEGFR1 protein localized in the blood vessels of the diabetic mouse retina (D) but was not detected in the non-diabetic control mouse retina (C). GCL: ganglion cell layer; IPL: inner plexiform layer; INL: inner nuclear layer; OPL: outer plexiform layer; ONL: outer plexiform layer. Scale bar: 50μm. (E & F) Quantification of WB (E) and IF (F) showed expression of VEGFR1 protein is up regulated in the retinas of Akita diabetic mice compared to non-diabetic control mice.

The results were averaged from four Akita diabetic mice and four non-diabetic control mice (n=4).

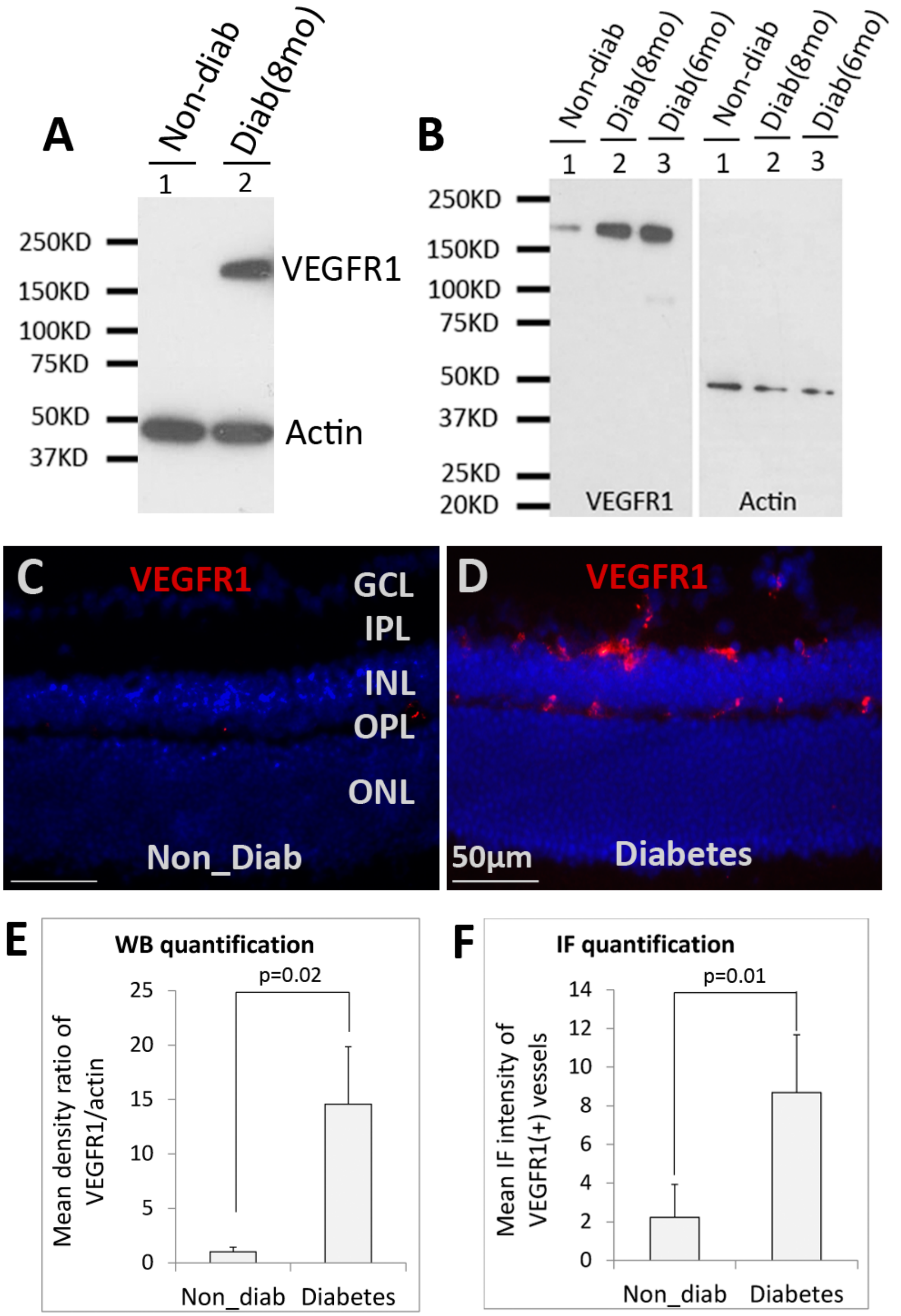

Supplement: Supplementary file 1 — Supplementary Table 1: The primer information for real-time PCR. The gene sequence was achieved from Genbank through the accession number. The oligonucleotide sequences were selected by primer3 software. Each primer pair amplified the unique PCR product as indicated. Supplementary Figure 1: The description was provided in the manuscript already. [file 605946.f1.pdf]
